# Lesions and pathogens found in pigs that died during the nursery period in five Danish farms

**DOI:** 10.1186/s40813-023-00319-9

**Published:** 2023-06-01

**Authors:** Kristiane Barington, Esben Østergaard Eriksen, Egle Kudirkiene, Karen Pankoke, Katrine Top Hartmann, Mette Sif Hansen, Henrik Elvang Jensen, Sophie Amalie Blirup-Plum, Benjamin Meyer Jørgensen, Jens Peter Nielsen, John Elmerdahl Olsen, Nicole Bakkegård Goecke, Lars Erik Larsen, Ken Steen Pedersen

**Affiliations:** 1 Department of Veterinary and Animal Sciences, Faculty of Health and Medical Sciences, University of Copenhagen, Ridebanevej 3, 1870 Frederiksberg C, Denmark; 2 Ø-Vet A/S, Køberupvej 33, 4700 Næstved, Denmark

**Keywords:** Herd health management, Histology, Immunohistochemistry, Microbiology, Pathology, Pigs, Real-time qPCR

## Abstract

**Background:**

Diagnosing and treatment of diseases in pigs are important to maintain animal welfare, food safety and productivity. At the same time antimicrobial resistance is increasing, and therefore, antibiotic treatment should be reserved for individuals with a bacterial infection. The aim of the study was to investigate gross and histological lesions and related pathogens in pigs that died during the nursery period in five Danish farms. In addition, high throughput, real-time qPCR monitoring of specific porcine pathogens in fecal sock and oral fluid samples were carried out to investigate the between-farm and between-batch variation in the occurrence of pathogens.

**Results:**

Twenty-five batches of nursery pigs from five intensive, indoor herds were followed from weaning (approximately four weeks) to the end of nursery (seven to eight weeks post weaning). Gross and histological evaluation of 238 dead and 30 euthanized pigs showed the highest prevalence of lesions in the skin, respiratory system, gastrointestinal tract, and joints. Gross and histological diagnoses of lung and joint lesions agreed in 46.5% and 62.2% of selected pigs, respectively. Bacteriological detection of *Escherichia coli*, *Streptococcus suis* or *Staphylococcus aureus* infections in joints, lungs and livers was confirmed as genuine infection on immunohistochemical staining in 11 out of 70 tissue sections. The real-time qPCR analysis of pooled samples showed that most pathogens detected in feces and in oral fluid in general followed the same shedding patterns in consecutive batches within herds.

**Conclusions:**

Gross assessment should be supplemented with a histopathological assessment especially when diagnosing lesions in the lungs and joints. Moreover, microbiological detection of pathogens should optimally be followed up by in situ identification to confirm causality. Furthermore, routine necropsies can reveal gastric lesions that may warrant a change in management. Real-time qPCR testing of fecal sock samples and oral fluid samples may be used to monitor the infections in the individual herd and testing one batch seems to have a good predictive value for subsequent batches within a herd. Overall, optimal diagnostic protocols will provide a more substantiated prescription of antibiotics.

**Supplementary Information:**

The online version contains supplementary material available at 10.1186/s40813-023-00319-9.

## Background

In order to optimize health and productivity, pig producers commonly adopt herd health programs advised by a veterinarian. Herd health management programs may facilitate reduced antimicrobial usage [1, 2]. This is important due to increasing antimicrobial resistance [3]. In Denmark, 76% of the veterinary-prescribed antibiotics are used in pigs [4], and the highest treatment intensity is during the weaning period [5]. One obvious way to decrease the amount of antibiotics used in pigs, is to secure that antibiotics are only used in situations where health problems are caused by susceptible bacterial pathogens. Therefore, there is a need to establish reliable diagnostic protocols for monitoring pathogens and diseases. Necropsies of dead pigs collected in herds may be used to select the correct treatment for the pen mates with similar clinical signs and extraction of information from porcine necropsy reports can provide information of value for animal health surveillance [6]. Discrepancy between clinical and postmortem diagnosed causes of death and diseases in humans and animals emphasize the importance of postmortem examinations [7–10]. In humans, total agreement between clinical and postmortem diagnoses has been reported to vary from 27.4 to 73.9% [7, 8, 11, 12]. Similarly, for dogs, cats and cattle total agreement between clinical and postmortem diagnoses vary between 36.2 and 39% [9, 10].

Pen-side necropsies are cheap and routinely carried out, however, they are restricted to gross evaluation which might be insufficient to reach a diagnosis. At least in humans, histopathological evaluation has shown to improve the interpretation of gross lesions [13–15].

Bacterial cultivation and PCR analysis of tissues sampled postmortem can be used for identification of pathogens. However, for both methods, detection of a specific pathogen does not necessarily imply that the pathogen is the cause of disease [16]. Instead, detection of bacteria could reflect agonal spread, contamination, commensal organisms, or postmortem bacterial translocation, i.e., endogenous bacteria such as *Escherichia coli*, *Klebsiella pneumoniae*, *Pseudomonas aeruginosa*, *Enterococcus* spp., *Clostridium* spp., and *Streptococcus* spp. multiply and migrate into the blood and tissues [16–18]. In addition, PCR only detects the expected known pathogens and not unknown or unexpected pathogens emerging in the herd [6]. Yet, in situ detection of a given pathogen in relation to pathological changes in the tissue clearly links it to genuine infection. However, the impact of viral infections that may predispose to secondary bacterial infection can not be disclosed histopathologically.

The presence of specific pathogens within a herd can be assessed by high-throughput real-time PCR analysis of fecal droppings collected from the pen floor using socks, and oral fluid samples collected with ropes [19]. Moreover, by continuous monitoring, specific pathogens such as swine influenza A virus, porcine circovirus type 2 (PCV2), *Brachyspira pilosicoli*, *Lawsonia intracellularis* and enterotoxigenic *E. coli* it is possible to detect changes in the pathogen burden within the herd over time [19]. However, pathogens detected in feces or oral fluid are not necessarily the cause of disease, since they may be present as commensals in both healthy and diseased animals [19–21].

Veterinarians commonly use historical diagnostic data from a herd in case of reappearance of similar clinical signs [22]. This might not be rational. For instance, fecal sock samples collected at diarrhea outbreaks in consecutive batches in Danish weaner units during a two-month period showed that shifts in the pathogen composition were very common [23]. Furthermore, according to Danish law laboratory diagnostic examinations of fecal or intestinal samples must be conducted once annually in pig farms using antibiotic batch medication for intestinal diseases. This may be too infrequent to rationally guide antimicrobial treatment schemes [24]. In contrast, it was recently reported that the pathogens followed the same shedding pattern in two outbreaks of post-weaning diarrhea in two Danish herds. In the first week after insertion to the nursery, rotavirus A was commonly detected in diarrheic pigs, while *E. coli-*associated cases dominated in the second week. Yet, it remains unknown whether this dynamic could reasonably be expected again in future batches inserted in the herds [25]. That is, should advising veterinarians survey every batch, or may pathogen patterns observed in one batch be extrapolated to future batches?

The aim of the study was to investigate gross and histological lesions and related pathogens in pigs that died during the nursery period in five Danish farms. In addition, high throughput, real-time qPCR monitoring of specific porcine pathogens in fecal sock and oral fluid samples were carried out to investigate the between-farm and between-batch variation in the occurrence of pathogens.

## Results

### Herds

Five intensive, indoor, production herds were included. In each herd, five consecutive batches of nursery pigs were followed from weaning (approximately four weeks) until the end of nursery (seven to eight weeks), i.e., 25 batches in total. Batch size, number of dead pigs, average, minimum and maximum body weight of dead pigs and other herd characteristics are listed in Table 1.

**Table 1:** Overview of herd characteristics. All herds were classified as specific pathogen free (SPF) with modifications. SPF classified herds (with no modifications) are free from* M. hyopneumoniae*,* A. pleuropneumoniae*, Porcine reproductive and respiratory syndrome virus, Toxigenic* Pasteurella multocida*,* Sarcoptes scabiei var. suis, Haematopinus suis* (lice) and* Brachyspira hyodysenteriae*

Herd	1	2	3	4	5
SPF Herd health status^a^	Myc, AP12	Myc, AP12	Myc	Myc	Myc, AP6, AP12
Number of sows	600	760	1350	0^b^	0^b^
Pen-places (number of nursery pigs)	3100	4300	3300	8600	2400
Pen-places (number of slaughter pigs)	0	2200	0	0	450
Pigs per batch	349–422	358–493	314–740	850–1000	245–429
No. of dead pigs (in five batches)^c^	32	84	26	110	36
Mortality rate, %	1.6	4.1^d^	1.1^d^	2.3	2.1
Average body weight of dead pigs (min–max), Kg	5.5 (0.6–26.7)	9.1 (1.9–33.6)	10.4 (3.0–31.6)	7.4 (2.3–31.8)	9.8 (1.9–31.1)
Feed type	Home mixed non-pelleted feed	Home mixed non-pelleted feed	Home mixed non-pelleted feed	Home mixed non-pelleted feed	Commer-cial pellet feed
Medicinal zinc oxide^e^	Yes	Yes	Yes	Yes	No

^a^Modified SPF status includes: Myc = not free from* Mycoplasma hyopneumoniae*, AP6 and AP12 = not free from* Actinobacillus pleuropneumoniae* serotype 6 and 12, respectively

^b^Remote sow unit

^c^Additional 6 pigs with unknown herd status due to lost eartags

^d^Mortality rate was calculated based on an estimated population size

^e^Medicinal zinc oxide was given in the first two weeks of the weaning period

### Prescription of antibiotics

In each herd, data regarding the amount of prescribed antibiotics were specified as treatments of respiratory disease, gastrointestinal (GI) disease, and locomotor system/central nervous system diseases, respectively. All herds treated against GI and locomotor system/central nervous system diseases while only herd no. 4, used antibiotics for treatment of respiratory disease (Table 2).

**Table 2:** Prescription of antibiotics

Herd	Study period	Respiratory disease (ADD/100 pigs/day)	GI disease (ADD/100 pigs/day)	Locomotor system and CNS diseases (ADD/100 pigs/day)	Total (ADD/100 pigs/day)
1	Apr–Jul 2019	–	13.7	0.1	13.8
2	Apr–Jul 2019	–	6.3	0.5	6.9
3	Sep–Jan 2019/2020	–	6.9	0.2	7.1
4	Aug–Nov 2020	3.6	5.1	1.6	10.3
5	Aug–Nov 2020	–	5.2	0.6	5.8

Average animal daily dose (ADD) per 100 pigs per day of prescribed antibiotics against respiratory disease, gastrointestinal (GI) disease and locomotor/ central nervous system (CNS) diseases per herd

### Fecal sock and oral fluid samples

**Fig. 1:**
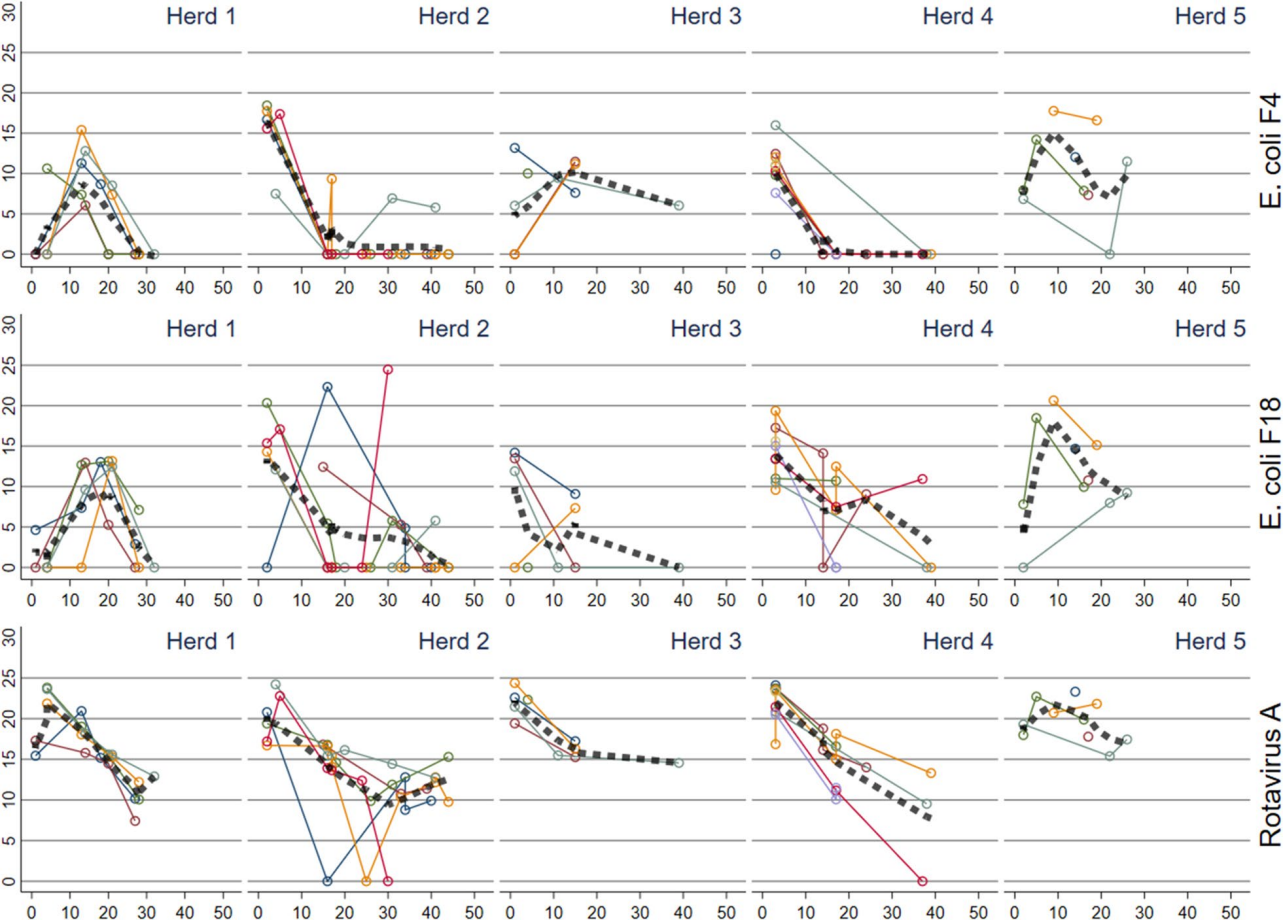
Reversed Ct values (30-Ct) for *Escherichia coli* F4 and F18, and rotavirus A detected in fecal sock samples in batches in five herds plotted against time (days) since insertion to the nursery. The thick dashed line represents a locally weighted scatterplot smoothing

**Fig. 2:**
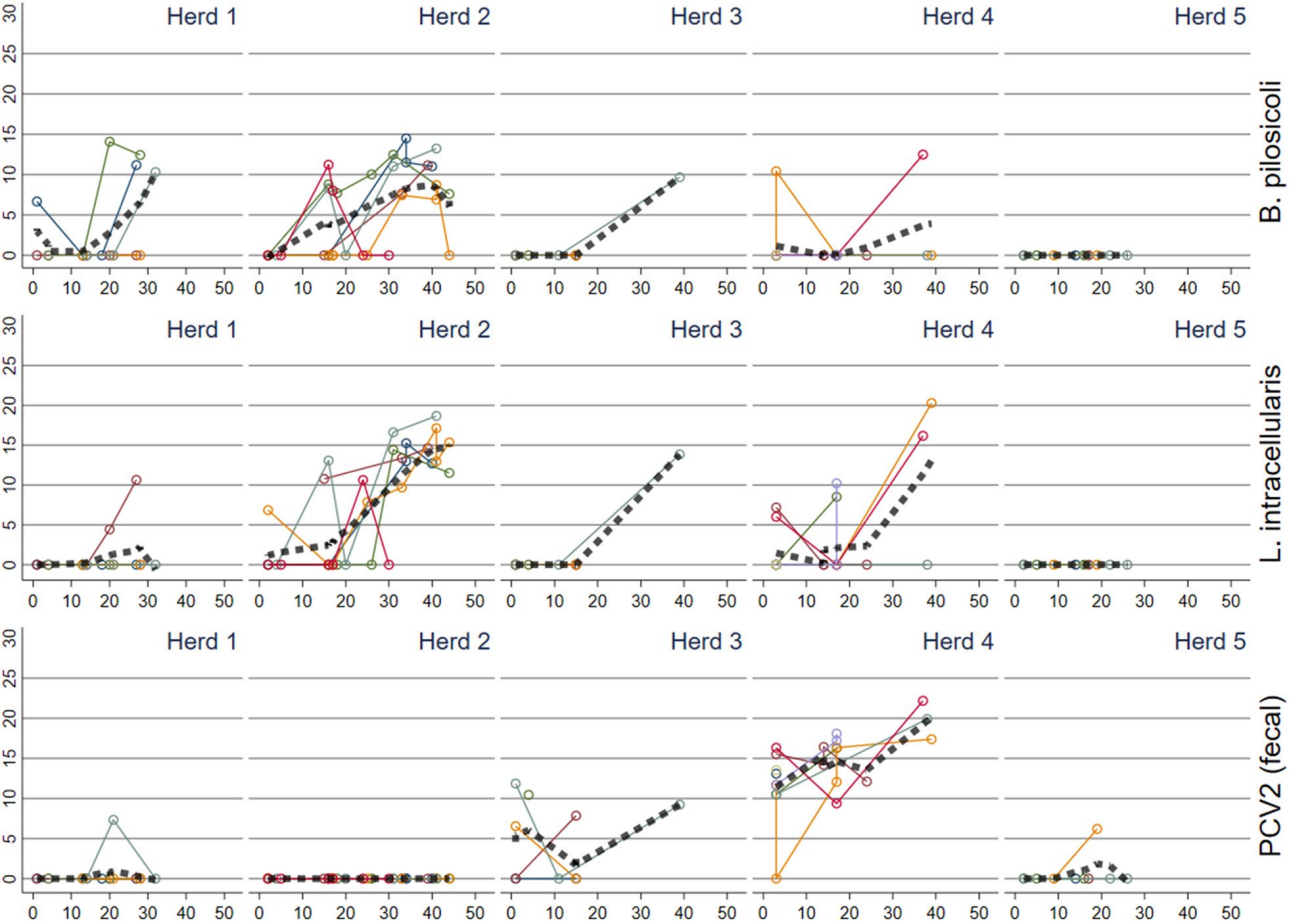
Reversed Ct values (30-Ct) for *Brachyspira. pilosicoli, Lawsonia. intracellularis,* and porcine circovirus 2 (PCV2) detected in fecal sock samples in batches in five herds plotted against time (days) since insertion to the nursery. The thick dashed line represents a locally weighted scatterplot smoothing

In all five herds, the enteric pathogens, *B. pilosicoli, E. coli* F4 and F18, *L. intracellularis,* and rotavirus A were shed following the same overall pattern (Figs. 1 and 2). Rotavirus A shedding peaked shortly after placement in the nursery unit and some shedding remained throughout the study period. Shortly thereafter, this was followed by shedding of *E. coli* F4 and F18. The pattern for *E. coli* was dissimilar between the herds since shedding peaked already at the first sampling in herd nos. 3 and 4, while the peak shedding was detected approximately 14 days after insertion in herd nos. 1 and 5 (Fig. 1). The shedding pattern of *E. coli* F4 and F18 varied somewhat between batches within herd no. 2. Later in the production period, *B. pilosicoli* and *L. intracellularis* started to dominate, even though herd no. 5 was an exception, as these two pathogens were not detected (Fig. 2). For each of the three pathogens, rotavirus B, C and H, the shedding patterns were somewhat similar across herds (Additional file 1).

In both the fecal samples and the oral fluid samples, PCV2 (Fig. 2, Additional file 2) showed a clear difference between herds, but a similar pattern between batches within the herds. PCV2 was either not detected or detected with low occurrence in herd nos. 1, 2 and 5, while herd no. 4 had a high and increasing occurrence in the older pigs. Interestingly for herd no. 3, batches were positive at or early after insertion to the nursery (Fig. 2).

**Fig. 3:**
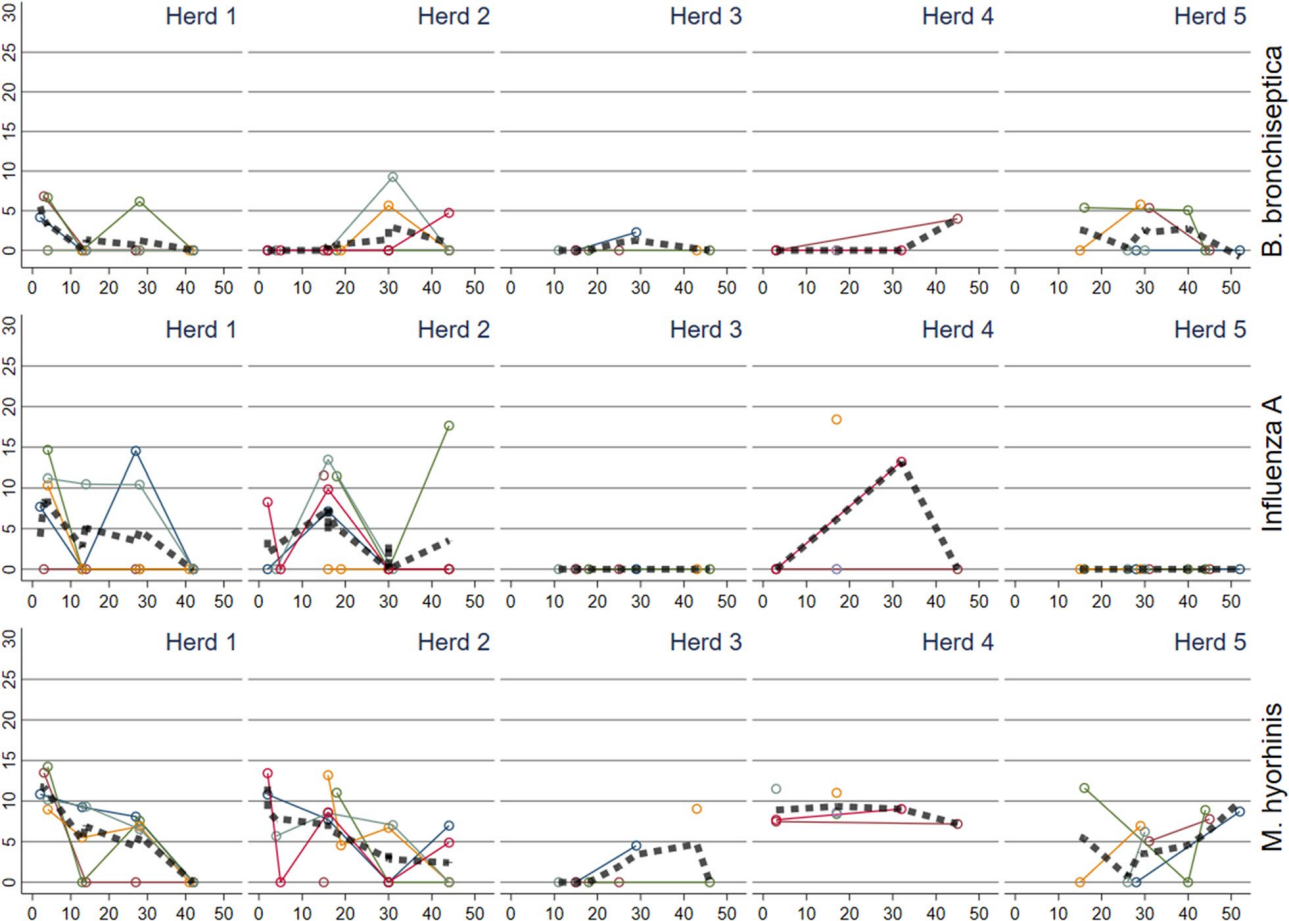
Reversed Ct values (30-Ct) for *Bordetella bronchiseptica,* influenza virus A, and *Mycoplasma hyorhinis* detected in oral fluid rope samples in batches in five herds plotted against time (days) since insertion to the nursery. The thick dashed line represents a locally weighted scatterplot smoothing

**Fig. 4:**
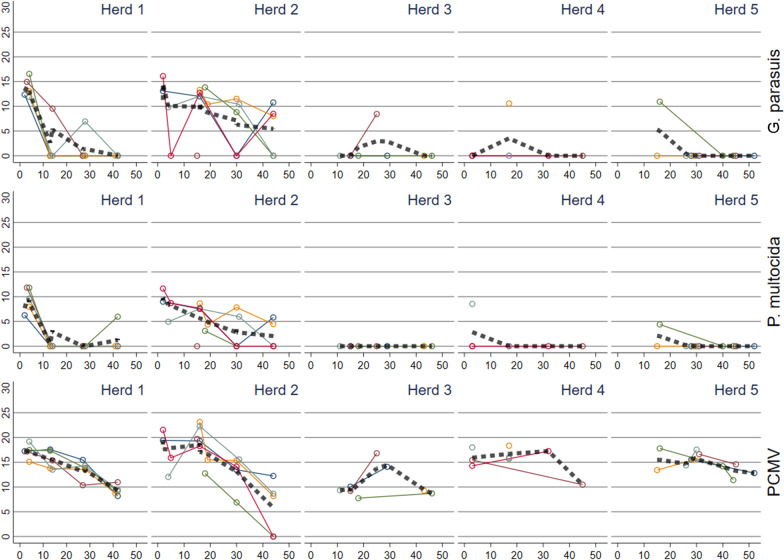
Reversed CT values (30-CT) for *Glaesserella parasuis, Pasteurella multocida,* and porcine cytomegalovirus (PCMV) detected in oral fluid rope samples in batches in five herds plotted against time (days) since insertion to the nursery. The thick dashed line represents a locally weighted scatterplot smoothing

For the pathogens detected in oral fluid, i.e., *Bordetella bronchiseptica*, influenza virus A, *Mycoplasma hyorhinis* (Fig. 3), *Glaesserella parasuis, Pasteurella multocida*, porcine cytomegalovirus (Fig. 4), *S. suis, Actinobacillus pleuropneumoniae* (Additional file 3)*,* and porcine circovirus 3 (PCV3) (Additional file 2) similar patterns were demonstrated, with different dynamics between herds. Within herds, the batches had comparable pathogen patterns showing either an increasing, decreasing, or constant level during the nursery period. For *Streptococcus suis* all herds had a constant occurrence during the nursery period and also a similar occurrence between herds (Additional file 3). PCV3 (Additional file 2) and *A. pleuropneumoniae* (Additional file 3) were not detected in two herds and detected sporadically in low levels in three herds. All samples were negative for *Mycoplasma hyopneumoniae* and porcine parvovirus.

### Gross and histopathological findings

During the study period, 288 dead and euthanized pigs were received for necropsy (Table 1). However, six and 14 pigs had to be discarded due to lack of identification by ear tags or severe autolysis, respectively. Therefore, 268 pigs (30 euthanized and 238 found dead) underwent a full necropsy, and tissue(s) were sampled for histological evaluation from 244 pigs (Additional file 4).

**Fig. 5:**
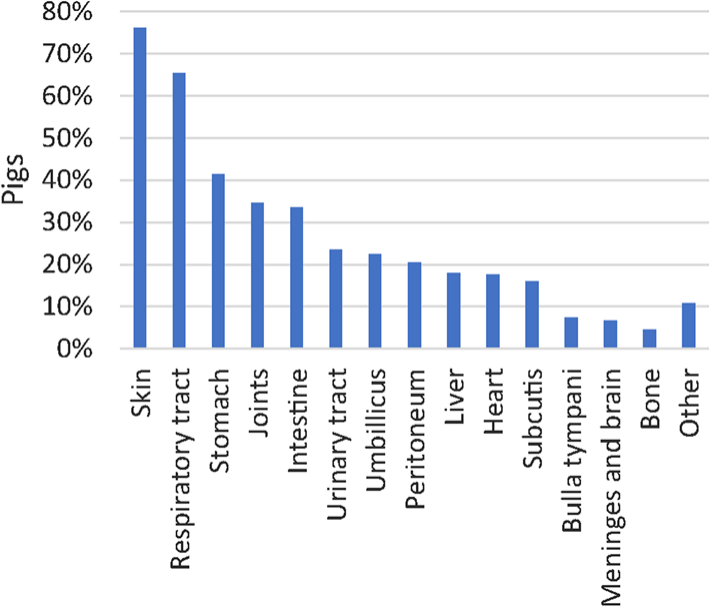
Prevalence of nursery pigs (n = 268) with lesions grouped according to the affected organ system. Lesions were registered at necropsy and at histological assessment when this was indicated, i.e., when gross evaluation alone was insufficient to obtain a diagnosis

Overall, lesions in the skin, respiratory tract, stomach, joints, and intestinal tract were most frequently seen (Fig. 5). However, the pattern differed between herds (Additional file 5).

#### Skin lesions

**Fig. 6:**
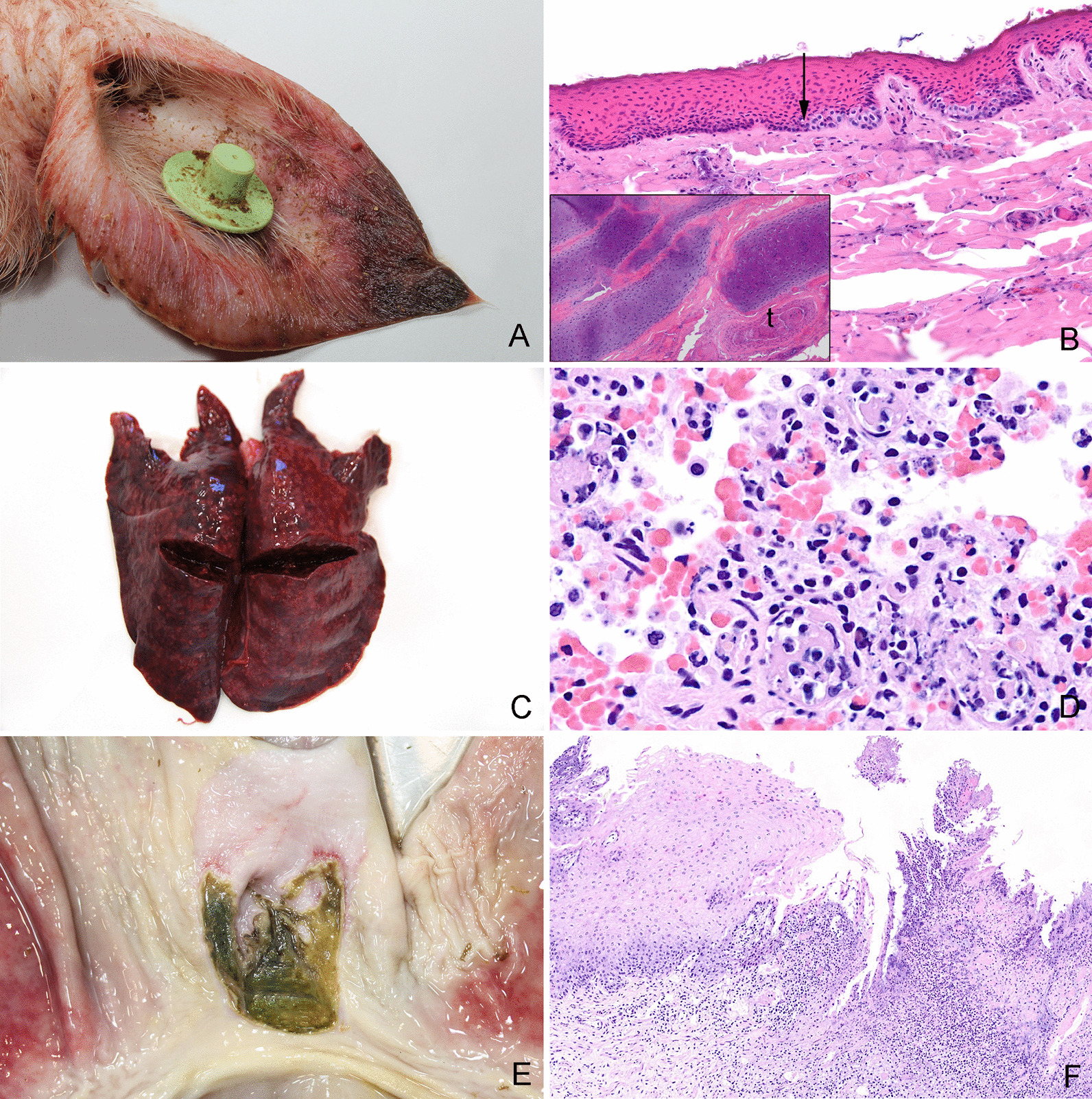
Gross and histological lesions in the skin, lungs and stomach. **A** Necrotic ulceration located at the apex of the pinna from a pig. Grossly, the necrotic tissue had a dark and dry appearance. **B** Tissue sample from a necrotic ear ulceration. Histologically, the lesion is characterized by coagulation necrosis of the epidermis. The arrow points out the transition between necrotic and viable cells in stratum basale. Moreover, fragmentation of the cartilage and thrombosis (t) were present (see insert), hematoxylin and eosin. **C** Lungs from a pig with peracute, embolic pneumonia. Apart from being heavy, no lesions could be palpated in the lungs. Therefore, the rib impressions on the caudal lobes were interpreted as interstitial pneumonia. However, the histological evaluation of the lungs revealed an embolic pneumonia (**D**). **D** Peracute embolic pneumonia. The tissue was sampled from the lungs shown in **C**. Histologically, the lung tissue was characterized by hyperemia, hemorrhage, foci of thrombosis and infiltration of neutrophilic granulocytes, hematoxylin and eosin. **E** Ulcerations and hyperkeratosis in the pars cardiaca of the stomach from a pig. **F** Tissue sampled from the pars cardiaca of the stomach shown in **E**. Histologically, the pars cardiaca is characterized by parakeratosis, ulceration, thrombosis, necrosis and infiltration of leucocytes, hematoxylin and eosin

Skin ulcerations were the most frequent type of lesions encountered (204 pigs out of 268) (Fig. 5). In total, 536 skin ulcerations were registered on the ears (52.6%), tail (17.4%), limbs (17.4%), body (5%), head (4.5%), and on umbilical outpouchings (3.2%). Ulcerations on the ears were located at the basis, apex or dorsal pinnae of 148 pigs. Severe ear ulcerations, i.e., ear ulcerations accompanied by necrosis/dry gangrene were registered in 111 pigs (Fig. 6A, Table 3). Moreover, 62 pigs presented with severe, necrotizing ulcerations with complete or partial sequestration of the tail including the coccygeal vertebrae. Histologically, the affected skin on the ears and tails was characterized by coagulation necrosis, and variable presence of thrombosis and inflammation (Fig. 6B).

**Table 3:** Skin ulcerations

	Herd 1 (n = 27) (%)	Herd 2 (n = 77) (%)	Herd 3 (n = 25) (%)	Herd 4 (n = 104) (%)	Herd 5 (n = 35) (%)	All (n = 268) (%)
Skin ulcerations (all body parts in total)	70.4	61.0	76.0	85.5	82.9	76.1
Necrotizing ear ulcerations	33.3	42.9	24.0	51.9	25.7	41.4
Non-necrotizing ear ulcerations	4	3	28	15	31	14
Necrotizing tail ulcerations	22.2	18.2	16.0	28.8	22.9	23.1
Non-necrotizing tail ulcerations	18.5	7.8	4.0	16.3	5.7	11.6

Pigs with grossly visible skin ulcerations in each herd and in total. Moreover, the percentages of pigs with ulcerations located on the ears and tails are specified as being necrotizing or non-necrotizing based on gross evaluation

#### Lung lesions

Lung lesions were found in 137 (51.1%) pigs based on a combination of gross and histological evaluation (Table 4). In 127 pigs, lung tissue was sampled and evaluated histologically to state the diagnosis (Table 5). In 59 out of 127 pigs (46.5%), the gross diagnosis and the histological diagnosis were identical. Especially interstitial pneumonias and acute embolic pneumonias were difficult to diagnose based on gross evaluation only (Fig. 6C, D, Table 5).

**Table 4:** Pigs with lesions in the respiratory tract

	Herd 1 (n = 27) (%)	Herd 2 (n = 77) (%)	Herd 3 (n = 25) (%)	Herd 4 (n = 104) (%)	Herd 5 (n = 35) (%)	All (n = 268) (%)
Rhinitis	0	1.3	4	1.9	8.6	3
Lung lesions	70.4	37.7	40.0	53.8	65.7	51.1
Bronchopneumonia	22.4	13.0	4.0	27.9	40.0	22.4
Embolic pneumonia	11.1	1.3	0	3.8	5.7	3.7
Interstitial pneumonia	3.7	1.3	0	1.9	2.9	1.9
Lung oedema	25.9	18.2	8.0	5.8	0	10.8
Other	7.4	3.9	28.0	14.4	17.1	12.3
Pleuritis	14.8	18.2	0	9.6	8.6	12

Pigs with lesions in the respiratory tract including rhinitis, bronchopneumonia, embolic pneumonia, interstitial pneumonia, lung oedema, other lung lesions and pleuritis. The diagnoses were based on gross evaluation supplemented by histological evaluation

**Table 5:** Agreement between gross and histological assessment of lung lesions

		Histology					
		Bronco-pneu	Embolic pneu	Interstitial pneu	Lung oedema	Other lesions	No lesions
Necropsy	Broncho-pneu	41 (32.3%)	3 (2.4%)	2 (1.6%)	5 (3.9%)	19 (15.0%)	2 (1.6%)
	Embolic pneu	0	1 (0.8%)	0	0	0	0
	Interstitial pneu	3 (2.4%)	3 (2.4%)	2 (1.6%)	9 (7.1%)	2 (1.6%)	3 (2.4%)
	Lung oedema	0	1(0.8%)	0	8 (6.3%)	2 (1.6%)	0
	Other lesions	4 (3.1%)	0	1 (0.8%)	7 (5.5%)	7 (5.5%)	2 (1.6%)
	No lesions	0	0	0	0	0	0

Agreement between gross and histological assessment of lung lesions in 127 pigs. Numbers and percentages in brackets of pigs diagnosed with bronchopneumonia, embolic pneumonia, interstitial pneumonia, lung oedema, other lesions, or no lesions in the lungs are presented

#### Stomach

Gross lesions in the stomach were present in 111 pigs and the most prevalent lesion was hyperkeratosis in pars cardiaca (Fig. 6E, F, Table 6).

**Table 6:** Lesions in the gastrointestinal tract

	Herd 1 (n = 27) (%)	Herd 2 (n = 77) (%)	Herd 3 (n = 25) (%)	Herd 4 (n = 104) (%)	Herd 5 (n = 35) (%)	All (n = 268) (%)
Gastric lesions	18.5	44.2	44.0	38.5	60.0	41.4
Hyperkeratosis NG	18.5	40.3	32.0	36.5	54.3	37.7
Ulceration NG	0	7.8	24.0	1.9	11.4	6.7
Ulceration G	0	2.6	4.0	3.8	5.7	3.4
Intestinal inflammation	40.7	31.2	48.0	26.9	42.9	33.6
Enteritis	33.3	19.5	36.0	19.2	37.1	24.6
Colitis/typhlitis/proctitis	14.8	19.5	20.0	16.3	25.7	18.7

Percentages of pigs with lesions in the gastrointestinal tract including gastric hyperkeratosis, gastric ulceration (NG = non-glandular part, G = glandular part), enteritis, and colitis/typhlitis/proctitis. The diagnoses were based on gross evaluation supplemented by histological evaluation

#### Intestinal tract

Inflammatory lesions in the intestinal tract were registered in 90 pigs. Of these, 44.4% had inflammatory lesions in the small intestine only, 26.7% had inflammatory lesions in the large intestine only, while 28.9% had inflammatory lesions in both (Table 6). Histologically, the lesions in 212 intestinal tissues (sampled from 105 pigs) were characterized as necrotizing (12.3%), proliferative (3.3%), hemorrhagic/hyperemic (29.7%) or non-inflammatory (54.7%).

#### Joints

Joint lesions were found in 93 pigs (Table 7). Of these, 77 pigs had lesions in more than one joint. In 82 pigs, synovial membrane was sampled and evaluated histologically to state the diagnosis (Table 8). In 51 out of 82 pigs (62.2%), the gross diagnosis and the histological diagnosis were identical (Table 8).

**Table 7:** Lesions in the joints

	Herd 1 (n = 27) (%)	Herd 2 (n = 77) (%)	Herd 3 (n = 25) (%)	Herd 4 (n = 104) (%)	Herd 5 (n = 35) (%)	All (n = 268) (%)
Joint lesions	11.1	24.7	12.0	49.0	51.4	35.1
Arthritis	7.4	22.1	12.0	40.4	17.1	26.1
Synovial proliferation	3.7	0	0	7.7	20.0	6.0
Other	0	2.6	0	1	14.3	3.0

Percentages of pigs with lesions in the joints including arthritis, synovial proliferation with no leucocyte infiltration, and other lesions including arthrosis, hemorrhage, and hyperemia. The diagnoses were based on gross evaluation supplemented by histological evaluation

**Table 8:** Agreement between gross and histological assessment of joints

		Histology			
		Arthritis	Synovial proliferation	Other	No lesions
Necropsy	Arthritis	42 (51.2%)	2 (2.4%)	1 (1.2%)	2 (2.4%)
	Synovial proliferation	11 (13.4%)	8 (9.8%)	4 (4.9%)	5 (6.1%)
	Other	4 (4.9%)	1 (1.2%)	1 (1.2%)	1 (1.2%)
	No lesions	0	0	0	0

Agreement between gross and histological assessment of joints from 82 pigs. Numbers and percentages in brackets of pigs diagnosed with arthritis, proliferation of synoviocytes, other or no lesions in the joints are presented. Joint lesions grouped as “Other lesions” included lesions such as arthrosis, hemorrhage and hyperemia

### Bacteriological culture from organs

**Fig. 7:**
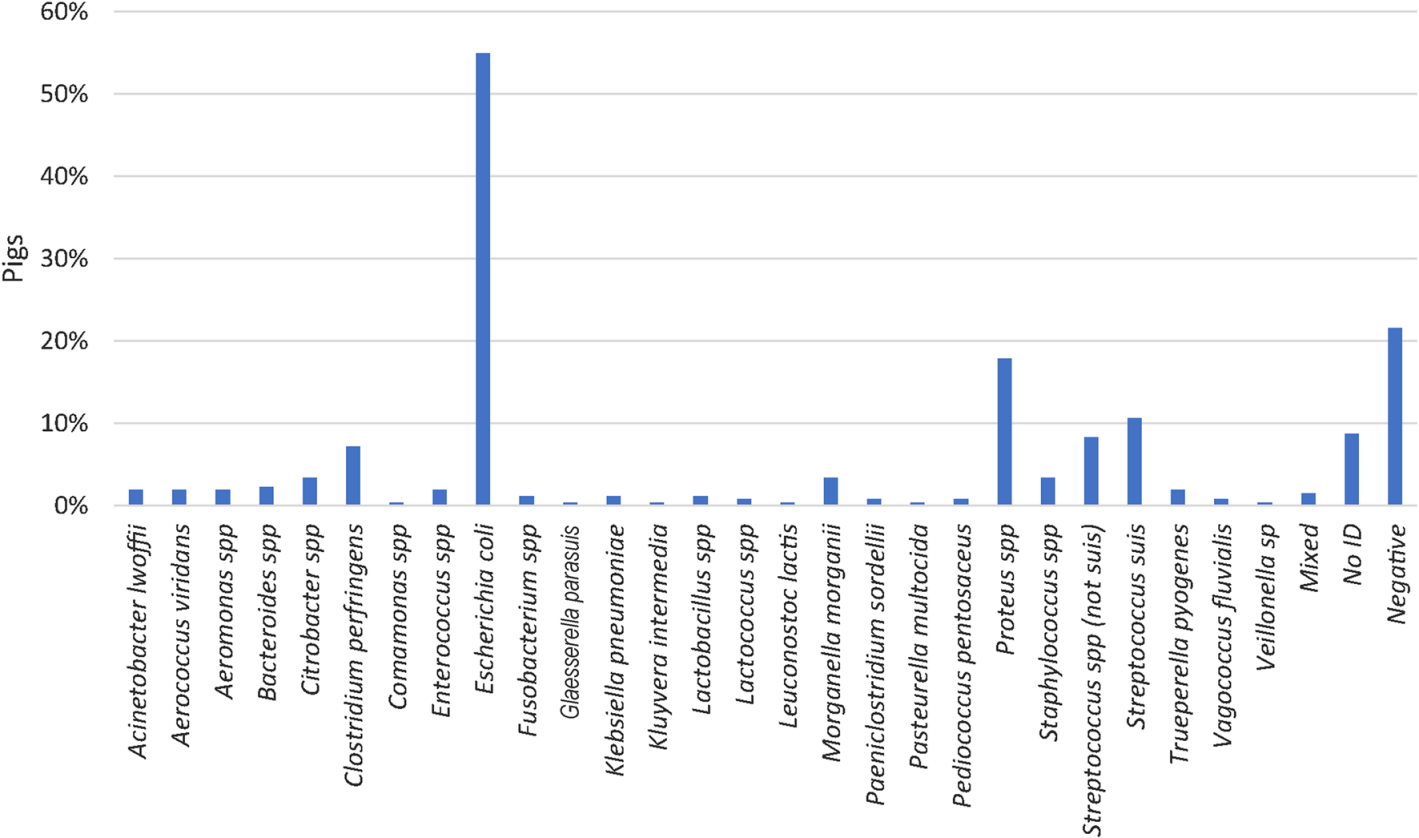
Prevalence of pigs (n = 264) positive for bacteria cultured from the liver and spleen. In total, 27 genus/species were identified across all five herds. A single species and multiple species were identified in 43.2% and 35.2% of the pigs, respectively

Bacteriological examination of the liver and the spleen was carried out in 264 pigs (Fig. 7). One or multiple species were identified in 43.2% and 35.2% of the pigs, respectively. In the remaining 21.6% the results were negative. In total, 27 genus/species were identified, of which *E. coli* was the dominating bacterium found in 54.9% of all pigs (Fig. 7). Pathogens detected varied relatively little between herds (Additional file 6). Bacteremia was defined as a positive cultivation result of a specific pathogen in both the liver and spleen. Bacteremia was present in 38.3% of the pigs. In 40.2% of the pigs the bacteria identified in liver and spleen were not identical or were identified as *Proteus* spp. The prevalence of species causing bacteremia are presented in Additional file 7.

**Fig. 8:**
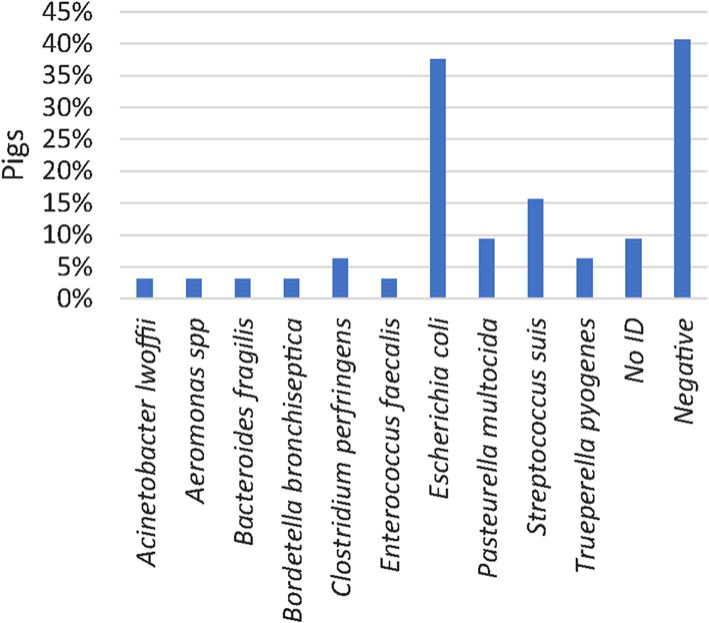
Prevalence of bacteria identified by microbiological examination of lungs with bronchopneumonia (n = 32 pigs). A single species or multiple species were identified in 31.3% and 28.1% of the pigs, respectively

**Fig. 9:**
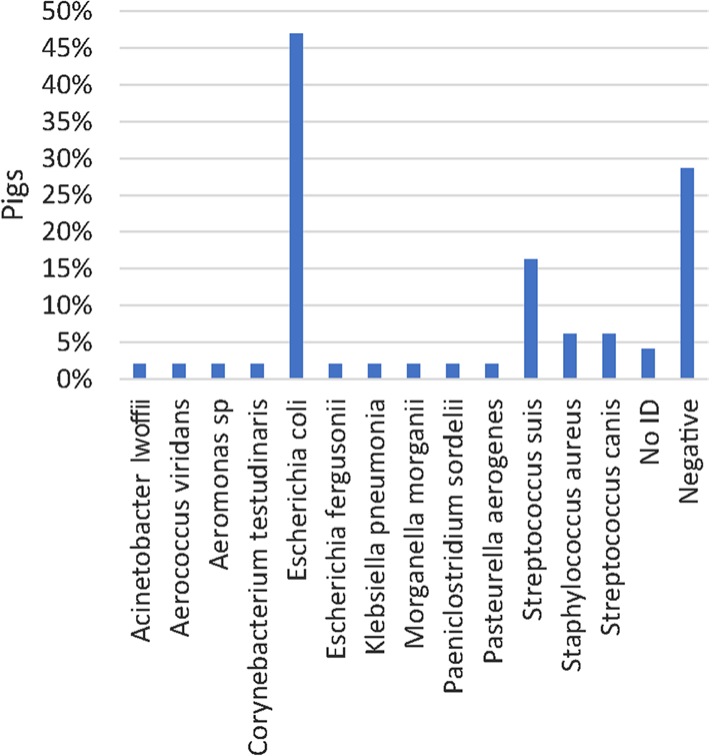
Prevalence of bacteria identified at microbiological examination of joints with arthritis and synovial hyperplasia (n = 49 pigs). A single species or multiple species were identified in 55.1% and 18.4% of the pigs, respectively

Microbiological examination of lungs with bronchopneumonia and joints with arthritis were carried out in 32 pigs and 49 pigs, respectively. In both tissues, *E. coli* was the most common species and found in 37.5% and 46.9% of the pigs, respectively (Figs. 8 and 9).

### Immunohistochemistry (IHC)

**Fig. 10:**
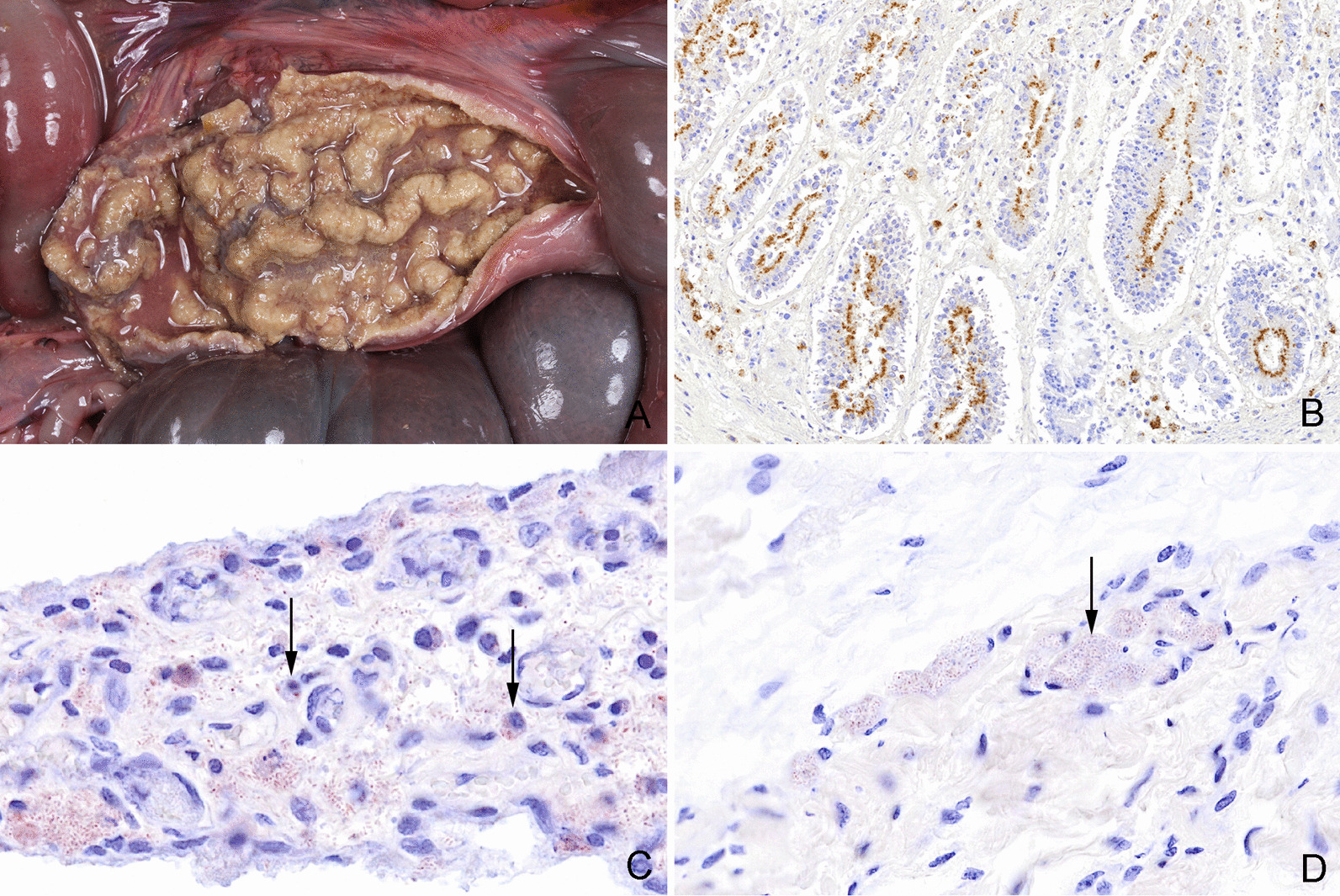
Gross and histological lesions in intestine and joints. **A** Necrotizing and proliferating ileitis in a pig caused by *Lawsonia intracellularis*. Similar lesions were present in the jejunum, caecum and colon. **B** Caecum from a pig with necrotizing, proliferative enterocolitis and typhlitis (Same pig as in Fig. 10A). Histologically, the proliferation of the crypt enterocytes and positive staining of *Lawsonia intracellularis* (stained brown) in the apical cytoplasm of the crypt enterocytes confirms the diagnosis, immunohistochemical detection of *L. intracellularis*. **C** Synovial villus with infiltration of macrophages. *Escherichia coli* (stained red) is present in the tissue and has been phagocytized by macrophages (arrows) indicating a genuine bacterial infection, immunohistochemical detection of *E. coli*. **D** Periarticular connective tissue from a joint with arthritis. *Escherichia coli* (stained red) is present in the vasculature (arrow). The absence of an inflammatory reaction near the bacteria is compatible with postmortem migration and proliferation of bacteria, immunohistochemical staining

*L. intracellularis* was identified in 8 of 18 pigs (seven in herd no. 2 and one in herd no. 5) with necrotizing and/or proliferative inflammation in one or more intestinal segments (Fig. 10A, B).

IHC staining for detection of *S. suis, Staphylococcus aureus* or *E. coli* was performed on samples of lung tissue (n = 14 pigs), synovial membrane (n = 27 pigs) and liver tissue (n = 23 pigs) that showed a positive cultivation result of these bacteria (Fig. 10C)*.* In six tissue samples both *S. suis* and *E. coli* were positive on cultivation, however, double infections were not confirmed by IHC staining. In none of the 23 livers, 1 of 21 joints, 2 of 11 lungs that were microbiologically positive for *E. coli,* the bacterium was identified by IHC and located in relation to inflammation (Fig. 10C). In the remaining IHC stained samples of liver, joints and lung tissue, *E. coli* was either not present (n = 38 samples) or present (n = 14 samples) but not related to inflammatory changes (Figs. 10D). Moreover, genuine *S. suis* and *S. aureus* infections (lungs and joints) were confirmed by IHC in 5 of 12 lesions and 3 of 3 lesions, respectively.

## Discussion

The present study demonstrated that lesions in dead and euthanized weaned pigs are predominantly located in the skin, the respiratory system, the joints, and in the gastrointestinal tract. In accordance with our findings, gastrointestinal and respiratory lesions were the most frequent findings in weaners necropsied at University of Bern, Switzerland from 2000 to 2011 [6]. Lung lesions were also dominating in pigs (from 10 weeks of age to “less than market weight”) slaughtered in the Czech Republic from 2010 to 2017 [26].

Lesions in the respiratory system, joints and intestines may have a bacterial etiology that usually can be treated with antibiotics. In herd no. 4, antibiotics were prescribed against respiratory disease, gastrointestinal disease and locomotor system/ CNS diseases which fits well with the high number of pigs with lesions in these organ systems. In the remaining four herds, antibiotics were prescribed for treatment of gastrointestinal disease and locomotor system/CNS diseases only, although the prevalence of pigs with e.g., bronchopneumonia was up to 40%. Under the assumption that lesions in dead and euthanized pigs to some extent reflect the general health in the herds, pigs in herd nos. 1, 2 and 5 might have benefitted from antibiotic treatment directed towards respiratory disease. However, it is unknown whether the respiratory diseases were clinically apparent.

Gross necropsies of dead and euthanized pigs can be carried out by veterinarians during herd visits. However, in some cases gross evaluation alone will not be sufficient to reach a diagnosis. Gross and histological diagnoses of lung or joint lesions were identical in 46.5% and 62.2% of cases. Similarly, in humans, histopathological assessment of tissue has major impact on the interpretation of lesions and determining the cause of death [13, 14]. In humans, the greatest discordances between gross and histological findings were reported for lesions in the lungs, kidney, liver, and heart [13, 14]. In the present study, diagnosing of interstitial pneumonia or acute/subacute embolic pneumonia required histological assessment. Moreover, in just 41 out of 72 pigs (56.9%) with grossly diagnosed bronchopneumonia, histological evaluation confirmed the diagnosis. In comparison, a gross diagnosis of bronchopneumonia in 279 human cases could be histologically confirmed in 69.2 to 73.8% of the cases [27]. The difference might reflect that the lungs of the nursery pigs were often relatively small making gross assessment of lesions more difficult or that some tissue samples were not representative for the gross lesions.

A limitation of the present study was that tissues were only sampled on indication, i.e., when gross evaluation alone was considered insufficient to obtain a diagnosis. However, this approach was chosen to imitate the reality where neither veterinarians nor veterinary pathologists perform systematic histological sampling due to time and cost limitations.

Arthritis diagnosed at gross inspection were histologically confirmed in 89.4% of the pigs, however more subtle lesions such as slight proliferation of the synovial membrane were more difficult to assess based on gross evaluation alone. This is in accordance with a study of experimentally induced arthritis in pigs in which some joints with histological changes indicative of arthritis showed no gross changes [28].

Due to postmortem intervals of up to 5 days, intestinal tissues were affected by autolysis which made it difficult to assess lesions grossly as well as histologically. Advanced autolytic changes in the intestinal tract are expected when the postmortem interval exceeds an hour or even less [29]. However, in routine necropsies, this would only be possible in freshly euthanized animals in which tissue is immediately sampled and fixated on site.

Gastric lesions were present in 18.5 to 60% of the pigs depending on the herds. The aetiology of gastric lesions is considered multifactorial and risk factors include low birth weight, small particle-sizes in the feed, housing environment and management [30–33]. The pigs in herd no. 5 had the highest prevalence of gastric lesions and were fed a commercial pellet feed, while in the other herds, pigs were fed a home mixed non-pelleted feed (Table 1). In accordance, pelleted feed has also been associated with severe gastric ulcers in slaughter pigs [30]. Gastric lesions have mostly been reported as a problem in finishing pigs [33, 34]. In two recent publications, however, gastric lesions were described in nursery pigs [31, 35]. In this study and in the studies by Peralvo et al. [31] and Blirup-Plum et al. [35] the most prevalent gastric lesion was hyperkeratosis in the pars cardiaca.

Skin ulcerations were registered in more than 70% of the pigs, and most ulcerations were located on the ears and tail. Apart from being potential portals of entry for bacterial infections, ulcerations are painful, at least in the acute stage, and thereby lowers animal welfare. Necrotizing ulcerations along the margin or at the tip of the pinna were observed in 41.4% of the pigs. Histologically, the ulcerations were characterized by coagulation necrosis with variable inflammatory response. Similar lesions have also been described in weaner pigs in other countries and have been named “Porcine ear necrosis syndrome” (PENS) [36–38]. The reported prevalence of PENS varies from of 4.4% in finisher pigs and between 11 to 35% in weaners [36, 38–40]. However, the prevalence of ear necrosis reported in previous studies is not directly comparable to the present study as our study population consisted of dead/euthanized pigs only. No definitive etiology has yet been discovered for PENS, however, the condition is speculated to be caused by a combination of multiple noninfectious and infectious agents such as mycotoxins, PCV2 and *Staphylococcus hyicus* [41, 42]. An association between PCV2 and PENS has been shown in some studies [38, 39]. In the present study, the level of PCV2 and percentage of pigs with necrotic ear ulcerations is doubtfully related. Herd no. 4 had the highest percentage of pigs with necrotic ear ulcerations (51.9%) and the highest level of PCV2 in the oral fluid samples. However, herd no. 2 only had a single PCV2 weak positive sample while still 42.9% of the pigs had necrotic ear ulcerations.

*E. coli* was the most prevalent species cultured from joints, lung tissue, spleens and livers (Figs. 7–9). In fact, *E. coli* was found in the liver or spleen of 55% of all dead pigs. Although, *E. coli* can cause inflammatory conditions in pigs [43, 44], it seems unlikely that more than half of the pigs could have had a genuine *E. coli* infection. Moreover, in only 1 of 21 joints, 2 of 11 lungs and none of the 23 livers that were microbiologically positive for *E. coli,* the bacterium was identified by IHC and located in relation to inflammation. In the remaining IHC stained samples *E. coli* was either not present or present but not related to inflammatory changes. Moreover, *S. suis* and *S. aureus* were detected by IHC in relation to inflammatory changes in 41.7% and 100% of the lesions in lungs and joints, respectively, that were otherwise positive on cultivation. In the present study, positive cultivation results not related to an infection may in part be explained by long postmortem intervals of up to 5 days before necropsy allowing bacteria to migrate from the mucosal surfaces and into the vessels and tissues. Moreover, false positive results could also be due to contamination of samples. In contrast, long postmortem intervals might influence the sensitivity of IHC detection of pathogens. The results indicate that culture positive results from internal organs of pigs investigated 1 to 5 days after death must not be done as they may not be a sign of infection but postmortem invasion.

Testing of pooled feces (sock samples) and oral fluid (rope samples) by real-time qPCR demonstrated that the dynamics were different between herds for most pathogens, but, interestingly quite constant between most batches within herds. This underlines the relevance of including different age groups in the diagnostic protocol, but also that the prediction for future batches in a herd may be acceptable, because of a quite constant occurrence from one batch to the next. This was in contrast to previous results using sock samples for enteric pathogens in batches one and two months apart, where a large variation between outbreaks of diarrhea was reported [23]. One explanation may be that the previous study investigated outbreaks of diarrhea during nursery while the current study used a fixed diagnostic protocol throughout the nursery period.

In conclusion, when necropsies are used as a diagnostic tool, they should be confirmed by a histopathological evaluation especially regarding disease in the lungs and joints. Moreover, necropsies can reveal herd problems, such as lesions in the stomach that may not clinically affect pigs but warrant changes in management to avoid worsening of the lesions as the pigs grow.

Microbiological detection of pathogens should optimally be followed up by in situ identification to confirm causality. Monitoring herds using real-time qPCR testing of fecal sock samples and oral fluid samples is relevant to demonstrate infections in the individual herd and testing one batch seems to have a good predictive value for future batches within the herd.

## Methods

### Herds

Five intensive, indoor, specific pathogen free (SPF) herds located on Zealand, Denmark were included in the study from April 2019 to January 2020 (herd nos. 1, 2 and 3) and from August 2020 to November 2020 (herd nos. 4 and 5). The herds were selected to represent Danish commercial intensive indoor herds in relation to feeding, housing, management, genetics, health-status and antimicrobial usage in nursery pigs. In addition, the herds needed to be located within a 2 h drive from Copenhagen, because samples and dead pigs were to be transported to University of Copenhagen. A list of herds fulfilling the previous criteria were identified from the portfolio of herds serviced by one specialized pig practice. Farmers on the list were contacted by telephone and asked for their willingness to participate in the study until five herds were included. In each herd, five consecutive batches were followed from weaning (approximately four weeks) and to the end of nursery (seven to eight weeks), i.e., 25 batches in total. All pigs were fed ad libitum and kept in a two-climate system, which comprises a partial slatted concrete floor and a covered lying area. Additional herd characteristics are listed in Table 1. Information regarding the amounts of prescribed antibiotics in the herds in the given study periods were available at VetStat, a database of all prescription drugs sold for the purpose of treating animals in Denmark (https://vetstat.fvst.dk/vetstat/).

### Fecal sock and oral fluid samples

Fecal sock samples were collected 1 and 14 days after weaning by using the procedure described by Pedersen et al. [23]. In each batch, only one sock sample was sampled at each time point and included all pens containing pigs from the batch. In case of an outbreak of diarrhea, a third fecal sock sample was collected prior to initiating treatment.

Oral fluid was collected 1, 14, 28 and 42 days after weaning by hanging a cotton rope in a pen for 30 min letting the pigs chew on the rope. After collection, the ropes were put in individual plastic bags and kept cool. In each batch, oral fluid was sampled from one randomly chosen pen at each time point.

All samples were kept at 5 °C for up to 96 h until they were stored at -20 °C until further analysis.

### Extraction of nucleic acids and high-throughput real-time polymerase chain reaction (PCR)

Nucleic acids were extracted from faecal sock and oral fluid samples using the extraction robot QIAcube HT (QIAGEN, Hilden, Germany) and the Cador Pathogen 96 QIAcube HT kit (Indical Bioscience, Leipzig, Germany) using the manufacturer’s instructions. Positive and negative (nuclease-free water; Amresco, Cleveland, OH) controls were included in each extraction. The nucleic acids were stored at − 80℃ until further analysis. Prior to high-throughput real-time PCR analysis, reverse transcription and preamplification were performed as described previously [35]. Target specific primers and probes against *B. pilosicoli*, *E. coli* F4 and F18, *L. intracellularis*, PCV2, porcine parvovirus, rotavirus A [45], and rotavirus B, C and H (primers not yet published) were applied on the faecal sock samples. For oral fluid samples target specific primers against *A. pleuropneumoniae, G. parasuis, M. hyopneumoniae, M. hyorhinis,* PCV2 and 3, *P. multocida,* porcine cytomegalovirus, *S. suis,* influenza virus A were applied [45].

The pre-amplified cDNA and DNA was stored at − 20 °C until high-throughput real-time PCR amplification, using the BioMark HD (Fluidigm, South San Franscisco, USA) and 192.24 dynamic array (DA) integrated fluidic circuit (IFC) system (Fluidigm) as previously described [45, 46].

### Gross examinations

From the selected 25 batches all dead and euthanized pigs were kept at 5 °C and transported twice a week (Mondays and Thursdays) to the University of Copenhagen for full necropsies according to the procedure described by Jensen [47]. Pigs were euthanized by the farmers based on their assessment, i.e., no criteria were given. After gross assessment, all livers and spleens were packed in plastic bags and submitted for microbiological evaluation that was carried out within 2 h. Moreover, selected lung lobes with bronchopneumonia and swabs from joints with arthritis were evaluated microbiologically.

### Histological examinations

Tissues and organs were sampled for histopathological examination on indication, i.e., when the gross evaluation alone was insufficient to obtain a diagnosis. Histological assessment of lesions was used to confirm or adjust the gross diagnosis. Moreover, lesions on the ears and the tail were sampled for histological characterization. Tissue for histopathological evaluation were immersion-fixed in 10% neutral buffered formalin for up to 5 days, then processed through graded concentrations of ethanol and xylene and finally embedded in paraffin wax. Tissue sections were cut at 4-5 µm and stained with hematoxylin and eosin.

### Bacteriological culture from organs

Using sterile tools and gloves the organs (liver, spleen and lung) were removed from the plastic bags and immersed into boiling water for up to 8 s depending on the size of the organ. A cut was made in the organ and a 10 uL loop was used to take a sample for plating. Swabs from joints were plated directly. Each sample was plated on two blood agar plates (Oxoid CM0055 with 5% of calf blood), which were incubated for 48 h at 37 °C with reading after 24 h and 48 h, one plate in microaerophilic conditions (candle jar) and another plate in anaerobic conditions. Plates were observed for bacterial growth, and all colonies with different morphologies were then purified on new blood agar plates and the species were identified using matrix assisted laser desorption ionization-time of flight (MALDI-TOF) mass spectrometry identification with VITEK®_MS RUO instrument (bioMérieux, Marcy l’Etoile, France) and CHCA matrix solution (Vitek® _MS-CHCA, bioMérieux SA) according to a standard procedure of the manufacturer. Spectra data was analyzed with the SARAMIS database.

### Immunohistochemistry (IHC)

*L. intracellularis:* Formalin fixed intestinal sections were IHC stained for *L. intracellularis*, when proliferative or necrotizing enteritis was present histologically. IHC staining was performed using a mouse monoclonal antibody (mAb) (Law1-DK/BIO 323, Bio-X Diagnostics) using the method described recently, [35].

Formalin-fixed sections of lung tissue and synovial membranes were IHC stained for *S. suis*, *S. aureus* or *E. coli* when animals either 1) presented with bronchopneumonia and were cultivation positive of *S. suis*, *S. aureus* or *E. coli* in lung tissue, or 2) presented with arthritis/synovial proliferation and were cultivation positive for *S. suis*, *S. aureus* or *E. coli*. Moreover, sections of liver tissue, that had already been sampled for histological evaluation, were IHC stained for *E. coli* if the bacteria were cultivated from the liver. Autolytic tissue sections (n = 6) were omitted.

For IHC identification of *S. suis* type 2 antigen, a specific polyclonal serum (article 22282 SSI Diagnostica, Denmark) was diluted 1:32,000 in tris-buffered saline (TBS) added bovine serum albumin 1% (BSA). For detection of *S. aureus*, a specific polyclonal antibody (Invitrogen PA1-7246,) was diluted 1:38,400 in TBS added 1% BSA. For detection of *E. coli* a specific polyclonal antibody (DAKO B0357) was diluted 1:56,000 in 5% normal swine serum and TBS.

The immunostainings were performed on 4–5 µm tissue sections by application of the Ultravision ONE Detection system horseradish peroxidase (HRP) (Epredia, TL-125-HLJ). First, the sections were dewaxed. No pre-treatment was performed for detection of *S. aureus*. For detection of *S. suis* tissue, sections were pre-treated in trypsin (pH 7.8) for 30 min at 37 °C. This was followed by blocking of endogenous peroxidase activity by 0.6% H_2_O_2_ in TBS for 15 min and blocking of unspecific binding by Ultra Protein Block for 5 min (Epredia). For detection of *E. coli,* blocking of endogenous peroxidase activity was followed by pre-treatment in Proteinase (P8038-16, Sigma-Aldrich) for 5 min. The tissue sections were then incubated with the primary antibody for approximately 20 h at 4 ºC. Ultravision HRP polymer (Epredia) was added for 30 min and AEC vector (AEC substrate kit SK-4200) for 10 min. Throughout the protocol, apart from the step between Ultra Protein blocking of unspecific binding and the application of the primary antibody, slides were washed in TBS, pH 7.6 for 2 × 5 min. Counterstaining was done in Mayer’s haematoxylin for 40 s (AMPQ002454.5000, VWR international) and the sections were rinsed in distilled water. Coverslips were mounted with glycerol-gelatine. Positive and negative controls were run simultaneously with each batch of staining. Negative controls for *S. suis*, *S. aureus* and *E. coli* included substitution of the primary antibody with a nonsense antibody (Rabbit immunoglobulin fraction, DAKO X0903), diluted in BSA/TBS at corresponding protein concentrations.

### Statistics

#### Mortality rate

The mortality rate was calculated as the percentage of dead and euthanized pigs out of the total population (the sum of pigs in all five batches). In herds nos. 2 and 3, the total number of pigs were missing for 2 batches and 1 batch, respectively. Therefore, an estimated total population was calculated by multiplying the average batch size by five. Then the estimated total population was used to calculate the mortality rate in each of the two herds.

#### Prescription of antibiotics

In the public register, VETSTAT [48] the prescribed antibiotics were available per month expressed as animal daily dose (ADD) per 100 pigs per day. In each herd an average ADD per 100 pigs per day was estimated for the specific study periods (4 to 5 months).

#### Real-time qPCR data

Ideally, a standard curve should be established to convert Ct values ​​into a copy number for the pathogens being investigated. We did not have this available, and thus we analyzed the raw Ct values. Reverse Ct values ​​(RCt) were estimated as:

$${\text{RCt }} = { 3}0 - {\text{Ct}}$$

The qPCR test was considered negative if there was no amplification curve or if the Cq value was below the detection limit. The detection limit of the PCR assays was between Cq value 26 and 28. For negative qPCR test, RCt was set to 0.

The reversed Ct-values were plotted batch-wise for each herd against the time since insertion in a scatterplot with connected dots. A locally weighted scatterplot smoothing [49] was added to suggest a trend across batches within herds.

#### Gross and histological data

Lesions registered at necropsy and histological assessment were grouped according to organ systems (Table 9) and the percentages of affected pigs were calculated for each herd and in total. A diagnosis based on histological assessment overruled the diagnosis based on the gross assessment. For the five most frequently affected organ systems percentages of pigs with specific diagnoses, e.g., bronchopneumonia, were estimated.

To assess the agreement between the gross and histological diagnoses in the lungs and joints, respectively, the percentages of pigs in which gross and histological diagnosis were similar were calculated.

**Table 9:** Overview of diagnoses grouped according to organ system/tissue

Organ/tissue	Lesions
Skin	Ulcerations on the ears, tail, limbs, body, head, and umbilical outpouchings
Respiratory tract	Rhinitis, pleuritis, bronchopneumonia, embolic pneumonia, interstitial pneumonia, lung oedema and other lesions such as hemorrhage, hyperemia, intravital atelectasis, bronchitis and hyperleukocytosis
Stomach	Ulceration, erosion, hyperemia, hyperkeratosis (non-glandular part)
Joint	Arthritis, synovial proliferation, arthrosis, hemorrhage, and hyperemia
Intestine	Enteritis, typhlitis and colitis further characterized as necrotizing and/or proliferative
Urinary tract	Glomerulonephritis, focal and embolic nephritis, interstitial nephritis, pyelonephritis, hydronephrosis, cysts, renal hemorrhage, cystitis, hydroureter, urethral inflammation
Peritoneum	Peritonitis
Liver	Hepatitis, perihepatitis, traumatic rupture/hemorrhage, congestive hepatopathy
Heart	Endo-, epi-, myo- and pericarditis, and endocardiosis
Subcutis	Subcutaneous oedema
Umbilicus	Omphalitis, abscess, or fibrosis in the umbilical area, hernias, eventrations and enterocystoma
Bulla tympani	Otitis media
Brain and meninges	Hydrocephalus, meningitis
Bone	Osteomyelitis, fractures, neoplasia, kyphosis/lordosis
Other	Serous fat atrophy, skin abscess, lesions in the tongue, spleen, reproductive system, and skeletal muscle

Lesions registered at necropsy and histological assessment were grouped according to organ systems. A diagnosis based on histological assessment overruled the diagnosis based on the gross assessment

#### MALDI-TOF data

The prevalence of each of the pathogens identified in the liver and spleen was estimated. Presence of a specific pathogen in both the liver and the spleen was defined as bacteremia. Moreover, the prevalence of pathogens in the lungs and joints of pigs diagnosed with bronchopneumonia and arthritis, respectively, was estimated.

## Supplementary Information

**Additional file 1:** Reversed Ct values for rotavirus B, C, and H detected in fecal sock samples in batches in five herds plotted against time since insertion to the nursery. The thick dashed line represents a locally weighted scatterplot smoothing. File format:.tif.

**Additional file 2:** Reversed Ct values for porcine circovirus (PCV) 2 detected in oral fluid rope samples and PCV2 and PCV3 detected in fecal sock samples in batches in five herds plotted against time since insertion to the nursery. The thick dashed line represents a locally weighted scatterplot smoothing. File format:.tif.

**Additional file 3:** Reversed Ct values for *Actinobacillus pleuropneumoniae,* and *Streptococcus suis* type 2 detected in oral fluid rope samples in batches in five herds plotted against time since insertion to the nursery. The thick dashed line represents a locally weighted scatterplot smoothing. File format:.tif.

**Additional file 4:** Number of pigs per herd from which tissue from organs/tissues were sampled and histologically evaluated. File format:.docx.

**Additional file 5:** Prevalence of lesions grouped according to organ system in dead or euthanized pigs in herd no. 1, no. 2, no. 3, no. 4, and no. 5. Lesions were registered at necropsy and at histological assessment when this was indicated, i.e., when gross evaluation alone was insufficient to obtain a diagnosis. File format:.tif.

**Additional file 6:** Prevalence of bacteria cultured from the liver and spleen in dead or euthanized pigs from herd no. 1, no. 2, no. 3, no. 4, and no. 5. File format:.tif.

**Additional file 7**: Prevalence of bacteria detected in pigs with bacteremia. Bacteremia was defined as the presence of a specific bacterium cultured from both the liver and the spleen. File format:.tif.

## Data Availability

The data supporting the conclusions of this article are included within the article and in the additional files. Moreover, additional datasets are available upon reasonable request.
