# Supplementary figures and images for: Lesions and pathogens found in pigs that died during the nursery period in five Danish farms
Source: Porcine Health Manag. 2023 Jun 1;9:26. doi: 10.1186/s40813-023-00319-9 (PMC10234047; doi:10.1186/s40813-023-00319-9)

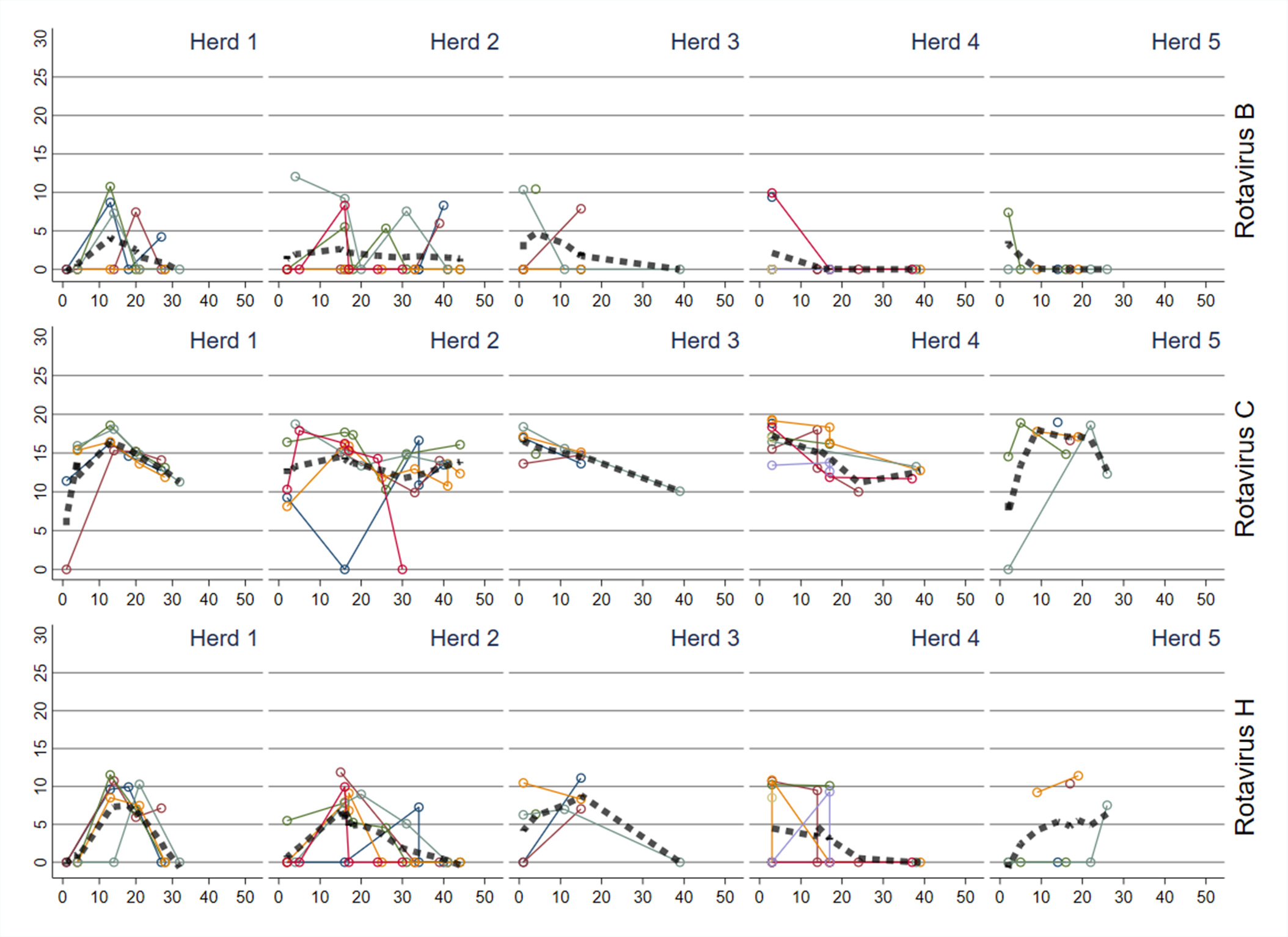

Supplement: Supplementary file 1 — Additional file 1: Reversed Ct values for rotavirus B, C, and H detected in fecal sock samples in batches in five herds plotted against time since insertion to the nursery. The thick dashed line represents a locally weighted scatterplot smoothing. File format:.tif. [file 40813_2023_319_MOESM1_ESM.tif]

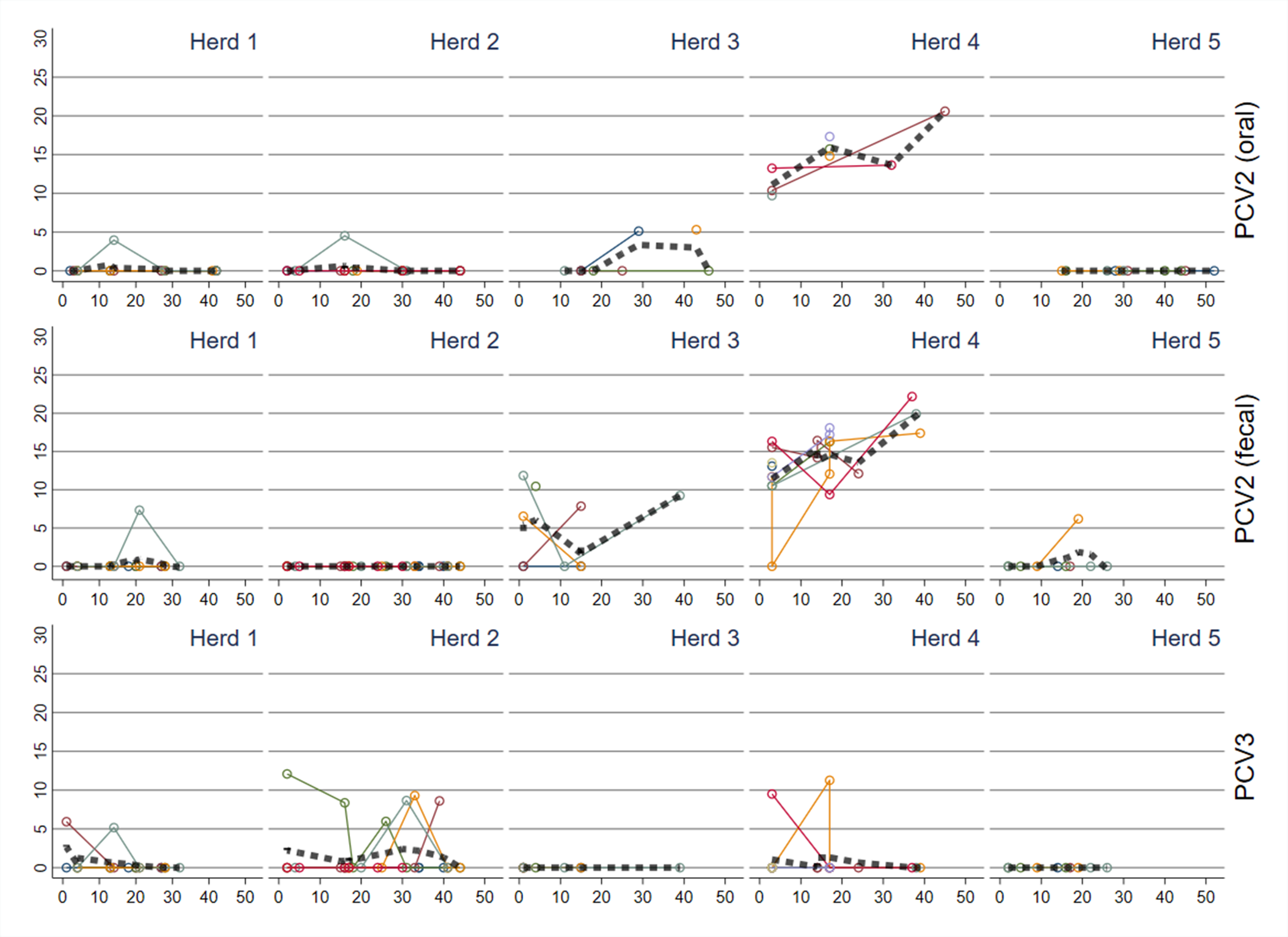

Supplement: Supplementary file 2 — Additional file 2: Reversed Ct values for porcine circovirus (PCV) 2 detected in oral fluid rope samples and PCV2 and PCV3 detected in fecal sock samples in batches in five herds plotted against time since insertion to the nursery. The thick dashed line represents a locally weighted scatterplot smoothing. File format:.tif. [file 40813_2023_319_MOESM2_ESM.tif]

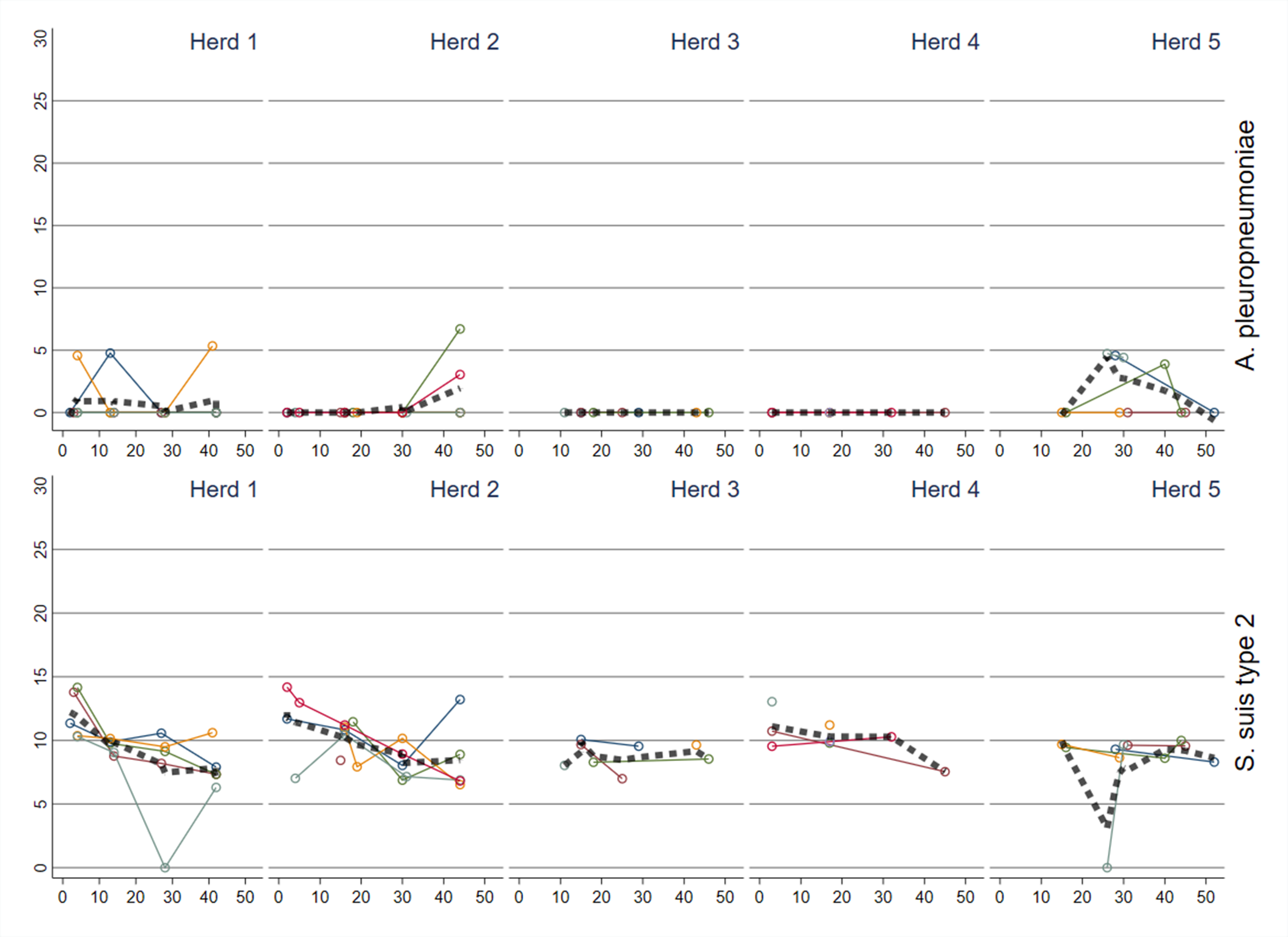

Supplement: Supplementary file 3 — Additional file 3: Reversed Ct values for Actinobacillus pleuropneumoniae, and Streptococcus suis type 2 detected in oral fluid rope samples in batches in five herds plotted against time since insertion to the nursery. The thick dashed line represents a locally weighted scatterplot smoothing. File format:.tif. [file 40813_2023_319_MOESM3_ESM.tif]

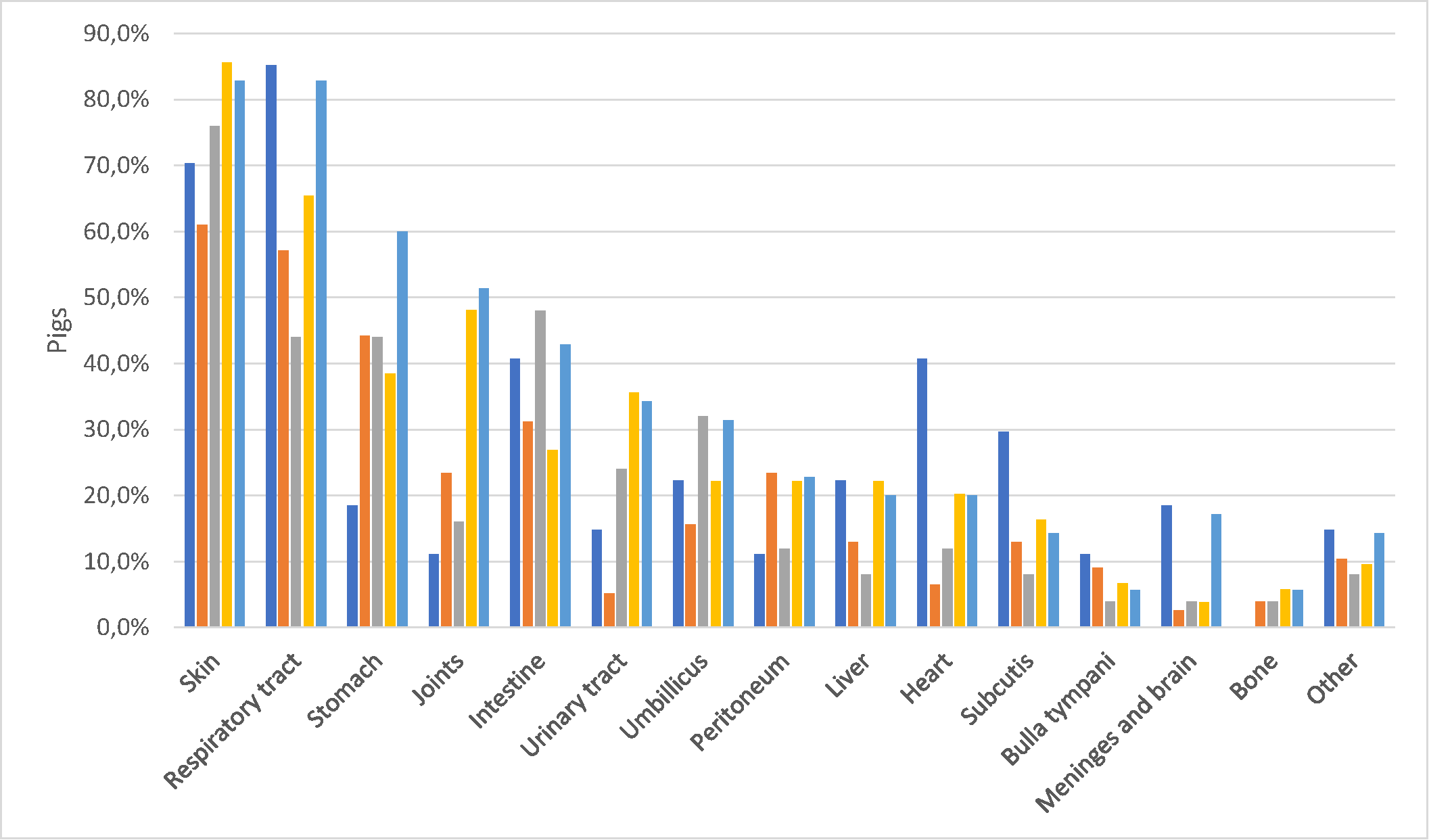

Supplement: Supplementary file 5 — Additional file 5: Prevalence of lesions grouped according to organ system in dead or euthanized pigs in herd no. 1, no. 2, no. 3, no. 4, and no. 5. Lesions were registered at necropsy and at histological assessment when this was indicated, i.e., when gross evaluation alone was insufficient to obtain a diagnosis. File format:.tif. [file 40813_2023_319_MOESM5_ESM.tif]

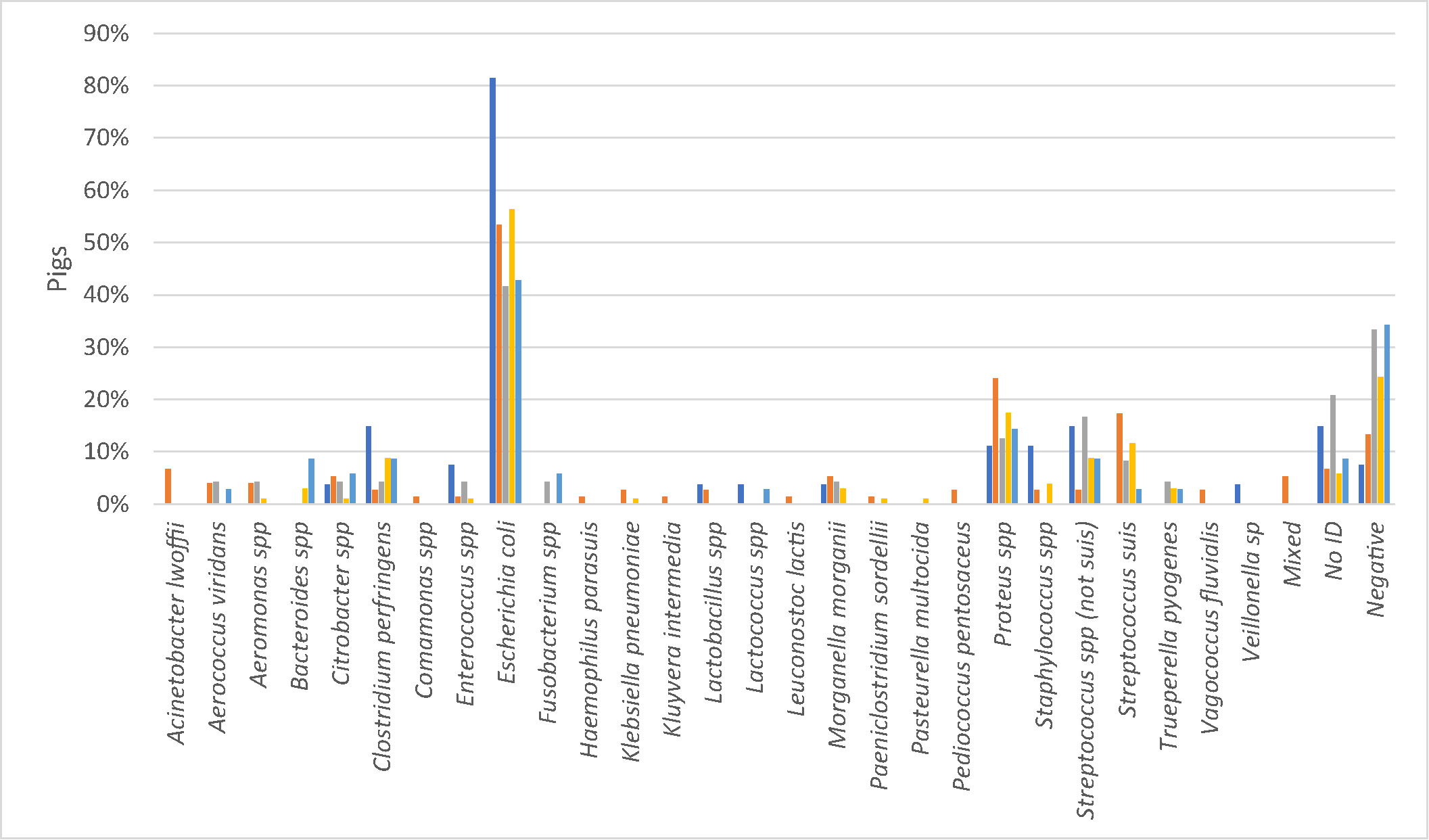

Supplement: Supplementary file 6 — Additional file 6: Prevalence of bacteria cultured from the liver and spleen in dead or euthanized pigs from herd no. 1, no. 2, no. 3, no. 4, and no. 5. File format:.tif. [file 40813_2023_319_MOESM6_ESM.tif]

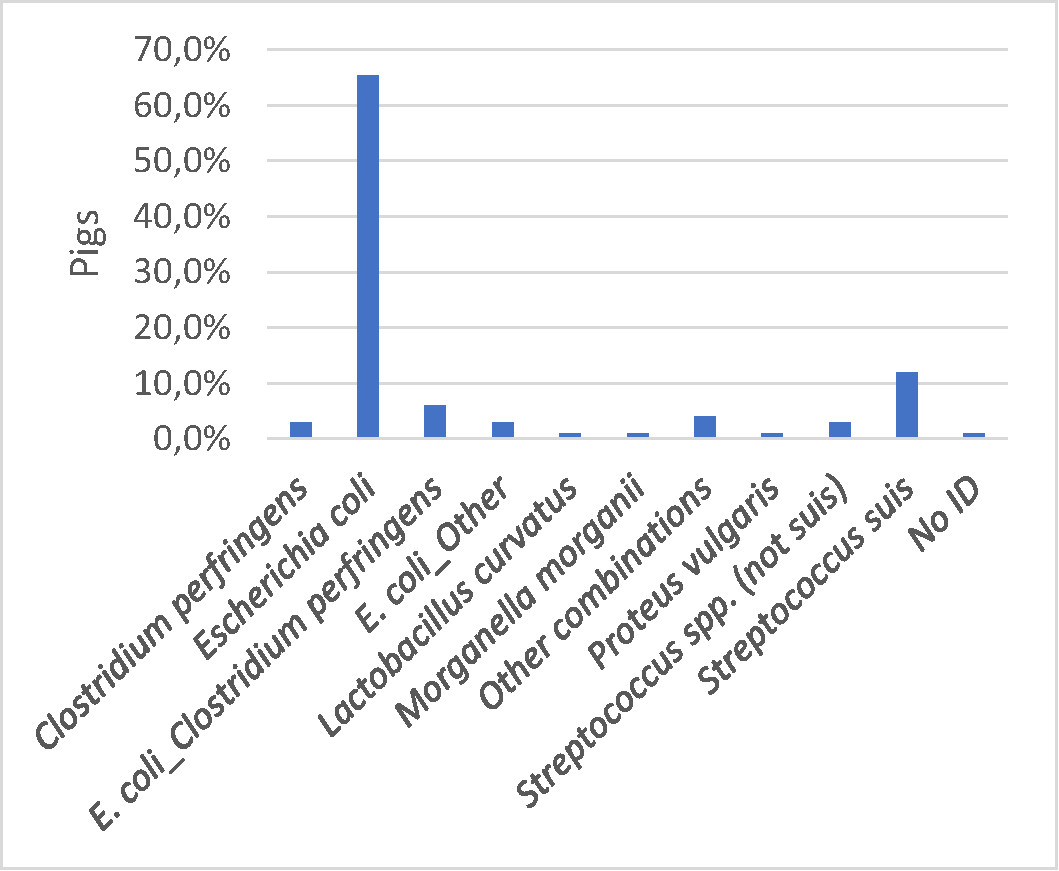

Supplement: Supplementary file 7 — Additional file 7: Prevalence of bacteria detected in pigs with bacteremia. Bacteremia was defined as the presence of a specific bacterium cultured from both the liver and the spleen. File format:.tif. [file 40813_2023_319_MOESM7_ESM.tif]
