# Supplementary material for: Lesions and pathogens found in pigs that died during the nursery period in five Danish farms
Source: Porcine Health Manag. 2023 Jun 1;9:26. doi: 10.1186/s40813-023-00319-9 (PMC10234047; doi:10.1186/s40813-023-00319-9)
Supplement: Supplementary file 4 — Additional file 4: Number of pigs per herd from which tissue from organs/tissues were sampled and histologically evaluated. File format:.docx. [file 40813_2023_319_MOESM4_ESM.docx]

**Additional file 4**

Number of pigs per herd from which tissue from organs/tissues were sampled and histologically evaluated.

| **Herd** | **1**  **(n=27)** | **2**  **(n=77)** | **3**  **(n=25)** | **4**  **(n=104)** | **5**  **(n=35)** | **All**  **(n=268)** |
| --- | --- | --- | --- | --- | --- | --- |
| Skin | 9 | 33 | 4 | 26 | 9 | 81 |
| Respiratory tract | 21 | 28 | 7 | 59 | 20 | 134 |
| Stomach | 3 | 19 | 7 | 18 | 8 | 55 |
| Joints | 4 | 16 | 1 | 47 | 19 | 87 |
| Intestine | 9 | 26 | 16 | 37 | 17 | 105 |
| Urinary tract | 3 | 1 | 0 | 22 | 4 | 30 |
| Peritoneum | 2 | 6 | 2 | 14 | 4 | 28 |
| Liver | 6 | 11 | 2 | 25 | 6 | 50 |
| Heart | 10 | 12 | 3 | 17 | 3 | 45 |
| Subcutis | 0 | 0 | 0 | 0 | 0 | 0 |
| Umbilicus | 3 | 5 | 0 | 6 | 3 | 17 |
| Bulla tympani | 3 | 7 | 0 | 7 | 1 | 18 |
| Brain and meninges | 1 | 0 | 1 | 0 | 2 | 4 |
| Bone | 0 | 2 | 0 | 3 | 1 | 6 |
| Other | 1 | 8 | 4 | 12 | 10 | 35 |
